# Body mass index is associated with health-related quality of life and disease characteristics in young adults with juvenile idiopathic arthritis

**DOI:** 10.1186/s12969-023-00931-7

**Published:** 2024-02-02

**Authors:** Anna-Kaisa Tuomi, Katariina Rebane, Ellen Dalen Arnstad, Lillemor Berntson, Anders Fasth, Mia Glerup, Troels Herlin, Hannu Kautiainen, Ellen Nordal, Suvi Peltoniemi, Marite Rygg, Veronika Rypdal, Marek Zak, Kristiina Aalto

**Affiliations:** 1 Pediatric Research Center, New Children’s Hospital, University of Helsinki and Helsinki University Hospital, Stenbackinkatu 9, P.O. Box 347, FIN-00029 HUS, 00290 Helsinki, Finland; 2 Department of Pediatrics, Levanger Hospital, Nord-Trøndelag Hospital Trust, Levanger, Norway; 3 Department of Clinical and Molecular Medicine, NTNU - Norwegian University of Science and Technology, Trondheim, Norway; 4 Department of Women’s and Children’s Health, Uppsala University, Uppsala, Sweden; 5 Department of Pediatrics, Institute of Clinical Sciences, Sahlgrenska Academy, University of Gothenburg, Gothenburg, Sweden; 6 Department of Pediatrics, Aarhus University Hospital, Aarhus, Denmark; 7 Kuopio University Hospital, Primary Health Care Unit Kuopio, Pohjois-Savo, Finland; 8 Folkhälsan Research Center, Helsinki, Finland; 9 Department of Pediatrics, University Hospital of North Norway and Pediatric Research Group, Tromsø, Norway; 10 Department of Clinical Medicine, UIT the Arctic University of Norway, Tromsø, Norway; 11 Helsinki University Central Hospital, HUS Inflammation Center, Rheumatology and University of Helsinki, Helsinki, Finland; 12 Department of Pediatrics, St. Olavs University Hospital, Trondheim, Norway; 13 Department of Pediatrics, Rigshospitalet Copenhagen University Hospital, Copenhagen, Denmark

**Keywords:** Body mass index, Juvenile idiopathic arthritis, Health-related quality of life, Disease activity, Disability

## Abstract

**Background:**

There is a growing interest concerning the relationship between obesity and several medical conditions and inflammation. Nevertheless, there is a lack of studies regarding body mass index (BMI) among patients with juvenile idiopathic arthritis (JIA). Our aim was to investigate the impact of BMI on health-related quality of life (HRQoL) measured with a 36-Item Short Form Survey (SF-36), disease activity, and disability in young adults with JIA.

**Methods:**

This study is a part of the population-based Nordic JIA cohort study. All newly diagnosed patients with JIA were recruited consecutively between 1997–2000 in specific regions in the Nordic countries. Patients in this sub-study were enrolled from 434 patients who attended their 18-year follow-up visit. Patients were classified according to the World Health Organization (WHO) into four groups based on their BMI. HRQoL, disease characteristics, disability, fatigue, sleep quality, physical activity, pain, comorbidities, and social status were assessed.

**Results:**

Three hundred fifty-five patients from the original study cohort were enrolled in this study and 72% of them were female. Mean age was 23.9 (± SD 4.4) years. A significant relationship was found between the JIA categories and BMI groups (*p* = 0.014). A significant relationship was also found between BMI and disease activity scores (DAS28) (*p* = 0.028), disability (*p* < 0.001), pain (*p* = 0.013), fatigue (*p* = 0.035), and sleep quality (*p* = 0.044). Moreover, a significant relationship between BMI and HRQoL regarding bodily pain (*p* = 0.010) and general health (*p* = 0.048) was revealed when adjusted for sex, age, and JIA subtype.

**Conclusion:**

We discovered that BMI was significantly related to HRQoL, disease activity, and disability. BMI deserves more attention considering the treatment options and outcome of JIA in young adults.

## Background

Juvenile idiopathic arthritis (JIA) [1] may cause various health problems during adulthood because of its chronic nature. JIA has been linked to other autoimmune diseases [2], mental health disorders [3], cardiovascular diseases [4], lower functional ability [5] and abdominal pain which is an important factor in the decreased quality of life [6].

Overweight and obesity have become increasing worldwide problems [7]. According to WHO, 39% of adults are overweight and 13% are obese [8].

The prevalence of overweight among patients with JIA is reported to be from 14.2% to 60% [9, 10]. Overweight and obesity have been associated with low-grade chronic systemic inflammation [11]. Obesity has been linked to rheumatoid arthritis (RA) [12] and especially to poor prognosis [13, 14]. To date, this topic is relatively little studied among young adults with JIA. Moreover, higher prevalence of overweight and obesity has been linked to autoimmune diseases among children and adolescents [15].

There is a lack of studies on body mass index (BMI) and JIA in young adults. Our aim was to study the impact of BMI on HRQoL and disease parameters among young adults with a JIA diagnosis.

## Methods

This study is part of the population-based Nordic JIA cohort study [16, 17]. All newly diagnosed patients with JIA were recruited consecutively between 1997–2000 in specific regions in the Nordic countries (Finland, Sweden, Norway, and Denmark). Patients in this sub-study were enrolled from 434 patients who attended their 18-year follow-up visit [16]. Seventy-five patients of the 434 were excluded because of missing data on either height, weight, or both. Consequently, 355 (82%) patients were eligible for this study.

BMI was categorized into four groups: BMI < 18.5 (underweight), BMI 18.5–24.9 (normal weight), BMI 25–29.9 (overweight) and BMI ≥ 30 (obesity) [18].

Children’s and adolescents’ (2–18 years) age- and sex-adjusted BMI was converted to ISO-BMI to correspond to an adult BMI by an age-appropriate factor. ISO-BMI < 17 (underweight), ISO-BMI 17–24.9 (normal weight) and ISO-BMI 25–29.9 (overweight) and ISO-BMI ≥ 30 (obesity) [19].

The SF-36 was used to evaluate the Health-Related Quality of Life (HRQoL) of the patients. The SF-36 includes eight domains measuring physical functioning, role limitation due to physical problems, bodily pain, general health perception, vitality, social functioning, role limitation due to emotional problems and mental health. Each domain is scored on a scale of 0–100. Zero represents the worst health status and 100 the best overall health status [20, 21].

The disease activity was evaluated by the Disease Activity Score in 28 joints (DAS28) [22]. DAS28 scales from 0 to 9.4; < 2.6 indicating remission, 2.6–3.2 low disease activity, 3.2–5.1 moderate disease activity and > 5.1 very high disease activity [23].

Functional ability was assessed by the Health Assessment Questionnaire (HAQ) [24]. The Wallace criteria were used to define remission [25]. Pain intensity was scored by the patient on a visual analogue scale (VAS), with 0 indicating no pain and 100 the worst possible pain.

Self-reported fatigue was assessed by the Fatigue Severity Scale (FSS, 0–7) [26]. Higher values indicate more severe fatigue. Information on sleep quality was evaluated with the Pittsburgh Sleep Quality Index (PSQI) comprising of seven categories: sleep duration, sleep disturbance, sleep latency, daytime dysfunction due to sleepiness, sleep efficiency, sleep quality, and use of sleep medication. The total score ranges from 0 to 21, with higher scores indicating poorer sleep quality [27].

Physical activity was assessed by asking questions on the frequency, intensity, and duration of exercise based on the Kasari-FIT index [28]. The score is 1–100, and a higher score indicates higher physical activity (0–12 insufficient amount, 13–36 moderate amount, 37–63 good amount and 64–100 excellent amount of exercise). The Kasari-FIT index was evaluated subsequently using the information collected at the 18-year study point. Exercise habits were assessed with the Frequency-Intensity-Time (FIT) Index [28].

Abdominal pain was classified into three categories according to the frequency: (1), never (2) seldom (one to three times a month) and (3) frequent (at least once a week).

Comorbidities, such as autoimmune thyroiditis and inflammatory bowel disease (IBD) were registered at the visit. Information on student and employment status as well as present medications were registered.

Approval for this study had been obtained from the local medical ethics committees. Informed consent was requested from all participants in accordance with the rules of the participating countries.

### Statistics

We present descriptive statistics as means with standard deviation (SD), and as counts with percentages. The hypothesis of linearity was tested using the Cochran–Armitage test, linear-by-linear, logistic models for categorical variables and analysis of variance (ANOVA) for continuous variables with an appropriate contrast. A possible nonlinear relationship between SF-36 dimensions and the Body Mass Index was assessed by using 3-knot-restricted cubic spline regression models; the models were adjusted for age, sex and diagnosis. The length of the distribution of knots was located at the 10th, 50th, and 90th percentiles. In the case of violation of the assumptions (e.g., non-normality) for continuous variables, a bootstrap-type method or Monte Carlo p-values (small number of observations) for categorical variables were used. The normality of variables was assessed graphically and by using the Shapiro–Wilk W test. Stata 17.0 (StataCorp LP, College Station, TX, USA) was used for the statistical analyses.

## Results

This study included 355 patients from the four Nordic countries. The number of female patients was 254 (72%) and male 101 (28%), and the mean age was 23.9 (± SD 4.4) years. Patients were assorted according to their BMI level. Disease characteristics and additional demographic data are presented in the Table 1.

**Table 1:** Disease characteristics according to BMI groups in the 18-year follow-up visit

	< 18.5 *N* = 21	18.5-24.9 *N* = 237	25.0–29.9 *N* = 59	≥ 30.0 *N* = 38	*P* value*
Female, n (%)	15(71)	173(73)	42(71)	24(63)	0.30
Age at onset, years, mean (SD)	4.0(3.2)	6.1(4.1)	7.8(3.8)	7.9(3.9)	< 0.001
Symptom duration, months, mean (SD)	8(16)	5(8)	7(9)	5(7)	0.95
ISO-BMI at onset, (z-score) mean (SD)	-1.51(3.69)	1.08(3.24)	2.39(2.93)	3.10(2.32)	< 0.001
JADAS at onset	2.2(3.6)	2.0(3.6)	1.9(3.3)	2.0(2.8)	0.80
Age	20.8(4.0)	22.9(4.4)	24.7(4.2)	25.2(4.3)	< 0.001
Disease duration	16.5(2.9)	16.7(1.6)	16.6(1.4)	17.1(1.0)	0.24
Subgroup of JIA, n (%)					0.014
Persistent oligoarthritis	9(43)	66(28)	10(17)	6(16)	
Extended oligoarthritis	3(14)	58(24)	10(17)	4(11)	
Polyarthritis, RF-positive	1(5)	2(1)	1(2)	2(5)	
Polyarthritis, RF-negative	5(24)	42(18)	10(17)	4(11)	
Psoriatic arthritis	1(5)	7(3)	4(7)	6(16)	
Enthesitis-related	0(0)	21(9)	8(14)	6(16)	
Systemic onset	0(0)	11(5)	2(3)	0(0)	
Undifferentiated	2(10)	30(13)	14(24)	10(26)	
DAS28	1.92(0.97)	1.83(0.86)	1.91(0.97)	2.26(1.01)	0.028
Tender joints 28	1.3(2.0)	0.6(1.7)	0.9(2.1)	1.9(4.3)	0.019
Swollen joints 28	0.5(0.7)	0.3(1.1)	0.5(1.5)	0.3(1.2)	0.92
Patients’ global, VAS	20.5(24.5)	14.7(22.7)	26.2(29.9)	29.3(33.8)	< 0.001
ESR	5.5(4.8)	6.7(6.8)	7.6(6.7)	12.3(9.6)	< 0.001
CRP	3.8(2.5)	2.8(3.7)	3.7(3.9)	7.2(8.3)	< 0.001
HAQ	0.15(0.35)	0.13(0.31)	0.33(0.59)	0.38(0.65)	< 0.001
Remission					0.17
No remission	14(67)	110(46)	24(41)	21(55)	
Remission on medication	0(0)	26(11)	3(5)	4(11)	
Remission off medication	7(33)	101(43)	32(54)	13(34)	
Pain, VAS	26.0(27.8)	15.8(22.0)	24.5(27.7)	28.1(29.9)	0.013
Fatigue total, mean (SD)	3.0(1.6)	3.1(1.5)	3.5(1.7)	3.6(1.6)	0.035
PSQI, mean (SD)	5.4(2.7)	5.5(3.4)	6.0(4.1)	6.8(4.1)	0.044
Kasari FIT-index	29.0(20.9)	33.9(23.2)	35.5(21.8)	28.8(19.2)	0.68
Abdominal pain					< 0.001
No	17(81)	197(83)	42(71)	24(63)	
Sometimes	3(14)	25(11)	8(14)	3(8)	
Always	1(5)	15(6)	9(15)	11(29)	
Comorbidities					
Hypothyreosis	0(0)	6(3)	2(3)	0(0)	0.78
IBD	2(10)	7(3)	1(2)	1(3)	0.30
Social status					0.004
Student	10(48)	119(50)	27(46)	9(24)	
Employed	6(29)	108(46)	29(49)	22(58)	
Unemployed	3(14)	5(2)	1(2)	1(3)	
On pension	2(10)	5(2)	2(3)	6(16)	

*ISO-BMI* Body Mass Index

*JADAS* Juvenile Arthritis Disease Activity Score

*JIA* Juvenile idiopathic arthritis

*RF* Rheumatoid Factor

*DAS28* Disease Activity Score 28

*ESR* Erythrocyte Sedimentation Rate

*CRP* C-reactive protein

*HAQ* Health Assessment Questionnaire

*VAS* Visual Analogue Scale

*PSQI* Pittsburgh Sleep Quality Index

*IBD* Irritable Bowel Disease

^*^P value for linearity across the three BMI levels

### BMI

**Fig. 1:**
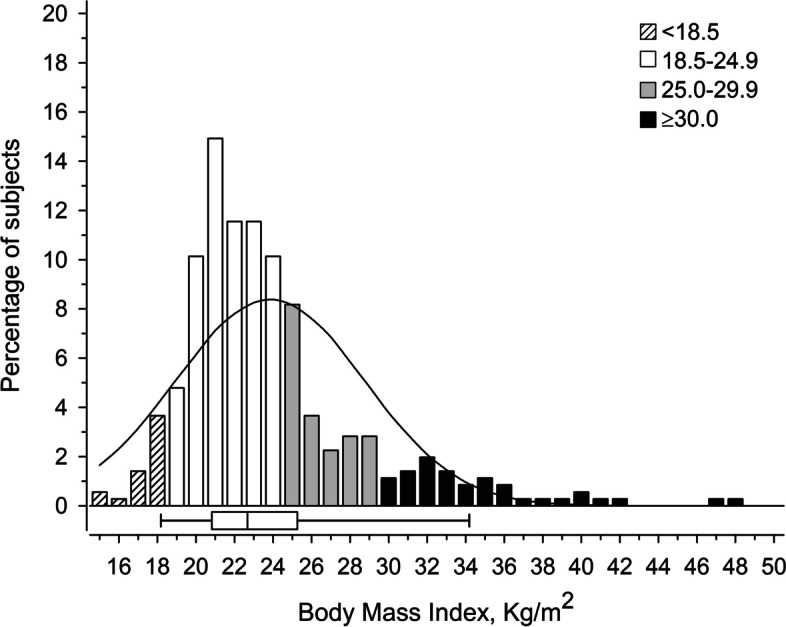
Distribution with normal curve overlay of BMI in the 18-year follow-up visit. Box-and-whiskers plot shows median and IQR, and whiskers indicate 5th and 95th percentiles

BMI was 23.8 (± 4.9); for females 23.5 (± 4.7) and males 24.6 (± 5.3) (Fig. 1). Six percent of the patients had BMI < 18.5 (underweight) and 11% of them had BMI ≥ 30 (obesity). The BMI categories (underweight, normal weight, overweight, and obesity) remained the same as they were at baseline (Table 1).

A significant relationship was found between BMI and onset ISO-BMI. Interestingly, we noticed that the BMI categories of both underweight and obese patients remained the same at the baseline and at the follow-up visit. At the baseline, the disease activity did not differ between the BMI groups.

### Clinical characteristics and outcome associated with BMI

Table 1 shows a significant association was found between the JIA categories and BMI groups. A significant relationship was found between BMI and disease activity already at the baseline. Thirty-four percent of underweight patients had persistent oligoarthritis, whereas 32% of overweight patients belonged to either the juvenile psoriatic arthritis (JPsA) or enthesitis-related arthritis (ERA) subgroup.

Higher BMI was related to disease activity according to DAS28 (*p* = 0.028), lower functional ability assessed with HAQ (*p* < 0.001) and to higher pain scores (*p* = 0.013).

Statistically significant relationships were found between BMI and fatigue (*p* = 0.035), poor sleep quality assessed with PSQI (*p* = 0.044), and abdominal pain (*p* < 0.001). Twenty-nine percent of patients in the obesity group reported frequent abdominal pain.

According to the Kasari FIT index, all patients in different BMI groups reported exercising moderately (Table 1).

No relationship between BMI and medication was found (Table 2). NSAIDs (Non-Steroidal Anti-Inflammatory drugs) were used by 59 patients, sDMARD (synthetic Disease Modifying Antirheumatic Drug) or bDMARD (biological Disease Modifying Antirheumatic Drug) by 103 patients, sDMARD by 70 and bDMARD by 74. In the sDMARD group, prednisolone was used by 10, methotrexate by 47, azathioprine by 4, hydroxychloroquine by 7, leflunomide by 4, sulfasalazine by 13 and cyclosporine by 3.

**Table 2:** Medication according to BMI groups in the 18-year follow-up visit

Medication	< 18.5 *N* = 21	18.5-24.9 *N* = 237	25–29.9 *N* = 59	≥ 30.0 *N* = 38	*P* value*
NSAIDs	1(5)	39(16)	10(17)	9(24)	0.15
sDMARD or bDMARD	7(33)	71(30)	15(25)	13(34)	0.99
sDMARD	5(24)	48(20)	9(15)	8(21)	0.66
bDMARD	5(24)	50(21)	11(19)	8(21)	0.76
prednisolone	1(5)	7(3)	2(3)	0(0)	0.47
methotrexate	2(10)	34(14)	5(8)	6(16)	0.99
azathioprine	1(5)	2(1)	0(0)	1(3)	0.99
hydroxychloroquine	0(0)	6(3)	1(2)	0(0)	0.99
leflunomide	1(5)	3(1)	0(0)	0(0)	0.16
sulfasalazine	0(0)	8(3)	4(7)	1(3)	0.60
cyclosporine	1(5)	1(0)	1(2)	0(0)	0.87

*NSAIDs* Non-Steroidal Anti-Inflammatory Drugs

*sDMARD* synthetic Disease Modifying Antirheumatic Drug

*bDMARD* biologic Disease Modifying Antirheumatic Drug

^*^P value for linearity across the three BMI levels

### Health-related quality of life associated with BMI

Statistically significant relationships were detected between BMI and role limitations due to physical health (*p* = 0.005), bodily pain (*p* = 0.021), general health perception (*p* = 0.008), and role limitations due to emotional health (*p* = 0.019) (Table 3). A statistically significant relationship was found between different BMI groups and the physical component score (*p* = 0.011) but not with the mental component score (*p* = 0.48).

**Table 3:** Quality of Life according to BMI groups in the 18-year follow-up visit

	< 18.5 *N* = 21	18.5-24.9 *N* = 237	25.0–29.9 *N* = 59	≥ 30.0 *N* = 38	*P* value*
SF-36					
Physical functioning	83(24)	90(18)	86(21)	85(20)	0.32
Role limitations due to physical health	83(30)	84(32)	76(39)	67(43)	0.005
Bodily pain	68(27)	76(23)	70(26)	64(27)	0.021
General health perceptions	67(25)	69(26)	63(26)	56(26)	0.008
Vitality	50(20)	60(24)	53(24)	52(25)	0.077
Social role functioning	76(27)	87(20)	82(25)	82(27)	0.48
Role limitations due to emotional health	81(36)	84(31)	77(37)	69(41)	0.019
Mental health	66(23)	76(17)	74(21)	74(20)	0.81
Summary scores					
Physical Component Score	51(9)	52(10)	50(11)	48(11)	0.011
Mental Component Score	45(11)	50(11)	48(13)	48(12)	0.48

*SF-36* 36-Item Short Form Survey

^*^P value for linearity across the three BMI levels

**Fig. 2:**
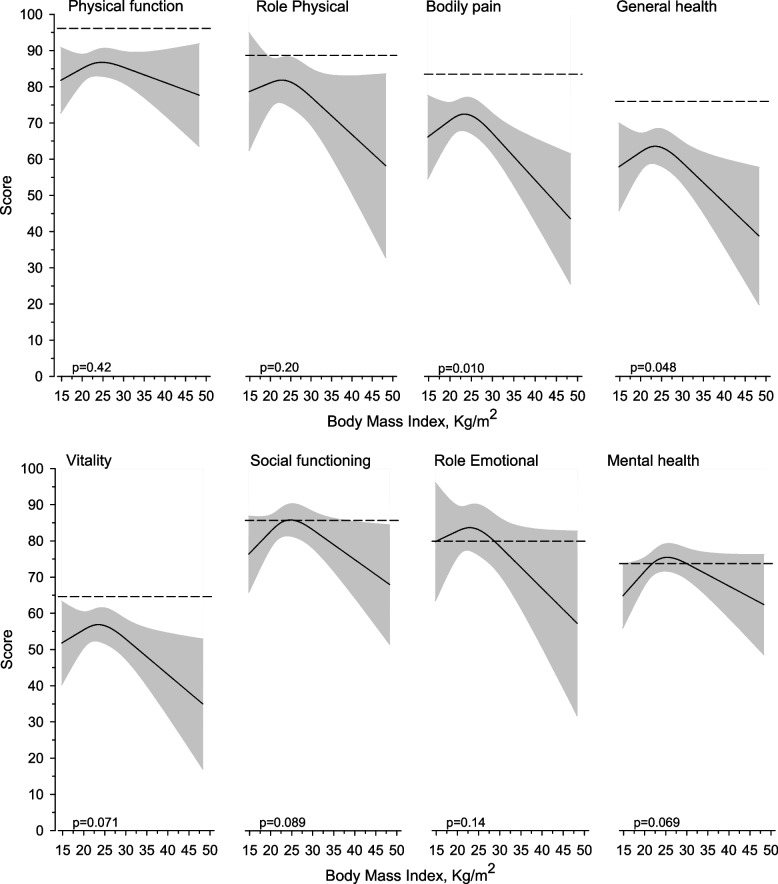
Relationships of dimensions of health-related quality of life as the function of the BMI in the 18-year follow-up visit. The curves were derived from 3-knot restricted cubic splines regression models. The models were adjusted for age, sex and diagnosis. The grey area represents a 95% confidence interval. Age- and gender-matched healthy controls from the study by Aalto et al. [21]shown by the dashed lines

When adjusted for age, gender and JIA categories, significant relationships were revealed between BMI and continuous bodily pain (*p* = 0.010) and general health (*p* = 0.048) (Fig. 2); patients with healthy BMI had better quality of life.

## Discussion

The main finding was that there was a significant relationship between BMI and HRQoL, highlighting that those patients with healthy BMI had better HRQoL. In addition, BMI at the 18-year follow-up visit was related to age, JIA category, disease activity, disability, pain, and student or employment status at the time of the follow-up visit, and to weight at diagnosis. Moreover, we revealed that BMI was related to fatigue and sleep quality.

Interestingly, we detected that the BMI categories of both underweight and obese patients remained unchanged at the baseline and at the follow-up visit. At the baseline, the disease activity did not differ between the BMI groups.

To our knowledge, the association between BMI and HRQoL has not been previously studied in young adults with JIA. We showed that in overweight/obese patients both the physical and mental dimensions of HRQoL, including bodily pain, general health perception, the role limitations due to physical or emotional health as well as vitality were impaired. Previous studies on HRQoL have shown that patients with JIA have more restricted functional ability, and they experience more pain compared to their healthy peers [29, 30]. On the other hand, it has been reported that adult patients with persistent oligoarthritis and ERA have a better HRQoL compared to the other JIA subgroups [31].

We found that BMI was associated with disease activity as measured by the DAS28. This association has been relatively little studied in JIA or in rheumatic diseases overall. A previous study suggests that obesity has a negative influence on the disease course and in the treatment response in JIA [32]. Excess adipose tissue can alter the pharmacokinetics of biological drugs and therefore diminish the treatment response [33]. This was also confirmed by a recent study in patients with psoriatic arthritis (PsA) [34]. Furthermore, ambivalent results have been revealed: no association between obesity and disease activity among patients with JIA in one study [35] and another study showed an association between underweight and higher disease activity [36].

Previously, it has been shown that growth and weight gain among children with JIA were comparable to the general population [37]. Children at risk of poor growth had systemic arthritis, uncontrolled disease and/or long-term use of corticosteroids [37]. A study from India showed that the weight, height, BMI, and growth velocity of children with JIA was reduced compared to controls [38]. RA patients that were overweight at the time of diagnosis were found to have a higher disease activity and they experienced more pain at the beginning of the disease [39].

In our study, we found an association between BMI and disability. This is consistent with a meta-analysis [40] showing that excessive body fat has an unfavourable effect both in the disease activity as well as in the functional ability of patients with RA, but others have shown contradictory results [41].

Obesity and JIA as individual factors have been linked to more pain in adolescence [42, 43]. Early self-reported pain in JIA has been found to predict persistent and unfavourable long-term disease outcome [44]. Previously, it has been shown that JPsA [45], ERA and undifferentiated JIA [46] patients experience more pain compared to other JIA subgroups. In our study, the patients in the obesity group reported significantly more pain than other BMI groups.

In previous studies obesity has been associated with higher inflammatory values in RA patients [47]. In our study we found that patients with higher BMI also had higher CRP and ESR. It remains to be clarified whether inflammatory values were elevated due to rheumatic inflammation or because of obesity. Earlier, it has been shown that high levels of inflammatory markers are related to adipose tissue and not to the disease activity in RA [47]. On the other hand, the central adiposity manifested in obesity may contribute to a persistent low-grade inflammation in patients with JIA [48]. In a Finnish study, although children with JIA had low disease activity, they had higher central adiposity compared to healthy controls [49].

Thirty-two percent of overweight patients in our study belonged to either the PsA or ERA subgroup. Previous studies are in line with our findings showing the association between obesity and different rheumatic disease categories in adults, such as PsA and RA [50, 51]. Obesity is among the most common comorbidities in patients with PsA [52, 53] and the prevalence of obesity was reported to be 44% [54]. Similarly, juvenile ERA patients were most likely to be overweight compared to other JIA categories [9].

Available data suggests that patients with JIA or RA experience more sleep disturbances and sleep less compared to their healthy peers, and they report more fatigue [55–57]. Adequate amount of sleep has many beneficial effects in children and adolescents [58]. Instead, short sleep duration is connected to excess fat accumulation, it impairs academic coping and is associated with lower QoL and emotional challenges [59]. Our study revealed that higher BMI was associated with fatigue and poor sleep quality, and, intriguingly, underweight patients had the best sleep quality, and they reported less fatigue. In line with our findings is a study that showed the association of overweight and obesity with poor sleep quality and short sleep duration [60]. Obesity, overweight and underweight were all associated with short sleep duration [61]. Moreover, poor sleep quality has other unfavourable effects, and it also causes constant low-grade inflammation [62]. Poor sleep together with depression is relatively common in arthritis patients [63].

Physical activity has many beneficial effects on general well-being, and mental and physical health [64–66]. Studies show that especially patients with JIA or RA benefit from physical activity [66], but JIA patients were found to be less active than their healthy peers [67]. A lower level of physical activity was found to be associated with overweight in patients with JIA [9, 65, 66]. From the very onset of JIA, children should be encouraged to take part in physical activity as the JIA diagnosis should not be a complete barrier to sports [68]. In this study we did not find a particularly low level of physical activity in any BMI group, and on average the levels of physical activity were eligible according to the Kasari-FIT [28].

Our study has several strengths. This study is long-term and prospective in its origin, and to our knowledge is the first study investigating the relationship between BMI and HRQoL in adult patients with JIA.

The minor limitation of the study is caused by the questionnaire-based and partially self-reported data by the patients including some missing data because of the long-term nature of the study. A limitation is also the absence of the control group. The study is based on a Nordic cohort and all new JIA cases were included, but it can be demanding to make broad generalizations based on these study results.

## Conclusions

Our novel findings suggest that BMI level should be acknowledged by healthcare professionals taking care of patients with arthritis. Based on our main findings we conclude that BMI and especially obesity impairs the HRQoL of adult patients with JIA. The patients should be encouraged to carry out physical activity together with weight control because of the versatile effect on general well-being. Based on our findings and previous data, we also recommend that sleep quality should be routinely assessed.

## Data Availability

The data are not publicly available for ethical and privacy reasons but are available by an appropriate permission request.

## Abbreviations

BMI: Body mass index
JIA: Juvenile idiopathic arthritis
HRQoL: Health-related quality of life
SF-36: 36-Item Short Form Survey
WHO: World Health Organization
DAS28: Disease Activity Score 28
RA: Rheumatoid arthritis
HAQ: Health Assessment Questionnaire
VAS: Visual Analogue Scale
FSS: Fatigue Severity Scale
PSQI: Pittsburgh Sleep Quality Index
FIT: Frequency-Intensity-Time
IBD: Inflammatory bowel disease
SD: Standard deviation
ANOVA: Analysis of variance
JPsA: Juvenile psoriatic arthritis
ERA: Enthesitis-related arthritis
NSAIDs: Non-Steroidal Anti-Inflammatory Drugs
sDMARD: Synthetic Disease Modifying Antirheumatic Drug
bDMARD: Biologic Disease Modifying Antirheumatic Drug
JADAS: Juvenile Arthritis Disease Activity Score
RF: Rheumatoid Factor
ESR: Erythrocyte Sedimentation Rate
CRP: C-reactive protein

## References

[1] Martini A, Prakken B, Albani S (2011). Juvenile idiopathic arthritis. Lancet.

[2] Pohjankoski H, Kautiainen H, Kotaniemi K, Korppi M, Savolainen A (2010). Autoimmune diseases in children with juvenile idiopathic arthritis. Scandinavian J Rheumatol Informa Healthc.

[3] Davis AM, Rubinstein TB, Rodriguez M, Knight AM. Mental health care for youth with rheumatologic Diseases - bridging the gap. Pediatric Rheumatology. BioMed Central Ltd.; 2017;15. 10.1186/s12969-017-0214-9 PMC5745617 29282086

[4] Avina-Zubieta JA, Thomas J, Sadatsafavi M, Lehman AJ, Lacaille D (2012). Risk of incident cardiovascular events in patients with rheumatoid arthritis: a meta-analysis of observational studies. Ann Rheum Dis.

[5] Carandang K, Vigen CLP, Ortiz E, Pyatak EA (2020). Re-conceptualizing functional status through experiences of young adults with inflammatory arthritis. Rheumatol Int.

[6] Rebane K, Tuomi AK, Kautiainen H, Peltoniemi S, Glerup M, Aalto K (2022). Abdominal pain in Finnish young adults with juvenile idiopathic arthritis. Scand J Gastroenterol.

[7] WHO. World Health Organization. 2023 . Obesity. Available from: https://www.who.int/health-topics/obesity#tab=tab_1. [Cited 2023 Aug 30].

[8] WHO. World Health Organization. 2023 . Obesity and overweigth. Available from: https://www.who.int/news-room/fact-sheets/detail/obesity-and-overweight. [Cited 2023 Aug 30].

[9] Schenck S, Niewerth M, Sengler C, Trauzeddel R, Thon A, Minden K (2015). Prevalence of overweight in children and adolescents with juvenile idiopathic arthritis. Scand J Rheumatol.

[10] Amine B, Ibn Yacoub Y, Rostom S, Hajjaj-Hassouni N (2011). Prevalence of overweight among Moroccan children and adolescents with juvenile idiopathic arthritis. Joint Bone Spine.

[11] Tilg H, Moschen AR (2006). Adipocytokines. Mediators linking adipose tissue, inflammation and immunity. Nat Rev Immunol.

[12] Dar L, Tiosano S, Watad A, Bragazzi NL, Zisman D, Comaneshter D (2018). Are obesity and rheumatoid arthritis interrelated?. Int J Clin Pract.

[13] Schulman E, Bartlett SJ, Schieir O, Andersen KM, Boire G, Pope JE (2018). Overweight, obesity, and the likelihood of achieving sustained remission in early rheumatoid arthritis: results from a multicenter prospective cohort study. Arthritis Care Res (Hoboken).

[14] Liu Y, Hazlewood GS, Kaplan GG, Eksteen B, Barnabe C (2017). Impact of obesity on remission and disease activity in rheumatoid arthritis: a systematic review and meta-analysis. Arthritis Care Res (Hoboken).

[15] Held M, Sestan M, Jelusic M. Obesity as a comorbidity in children and adolescents with autoimmune rheumatic diseases. 2023 ;43:209–19. 10.1007/s00296-022-05238-6. [Cited 2023 Jun 8]. 10.1007/s00296-022-05238-6 36394598

[16] Glerup M, Rypdal V, Arnstad ED, Ekelund M, Peltoniemi S, Aalto K (2020). Long-term outcomes in juvenile idiopathic arthritis: eighteen years of Follow-Up in the Population-based nordic Juvenile Idiopathic Arthritis Cohort. Arthritis Care Res (Hoboken).

[17] Berntson L, Gäre BA, Fasth A, Herlin T, Kristinsson J, Lahdenne P (2003). Incidence of Juvenile Idiopathic Arthritis in the nordic Countries. a population based study with special reference to the validity of the ILAR and EULAR Criteria. J Rheumatol.

[18] WHO. World Health Organization. 2023 . A healthy lifestyle - WHO recommendations. Available from: https://www.who.int/europe/news-room/fact-sheets/item/a-healthy-lifestyle---who-recommendations. [Cited 2023 Aug 30].

[19] Saari A, Sankilampi U, Hannila ML, Kiviniemi V, Kesseli K, Dunkel L (2011). New Finnish growth references for children and adolescents aged 0 to 20 years: Length/height-for-age, weight-for-length/height, and body mass index-for-age. Ann Med.

[20] Ware JE, Sherbourne CD (1992). The MOS 36-item short-form health survey (SF-36) I. conceptual framework and item selection. Med Care.

[21] Aalto A-M, Aro AR, Teperi J. RAND-36 as a measure of Health-Related Quality of Life. Reliability, construct validity and reference values in the Finnish general population. Helsinki; 1999.

[22] Prevoo MLL, Van ’t Hof MA, Kuper HH, Van Leeuwen MA, Van De Putte LBA, Van Riel PLCM (1995). Modified Disease Activity scores that include twenty-eight-joint counts. Development and Validation in a prospective longitudinal study of patients with rheumatoid arthritis. Arthritis Rheum.

[23] Fransen J, van Riel PLCM. The Disease activity score and the EULAR Response Criteria. Rheumatic Disease Clinics of North America; 2009;35. 10.1016/j.rdc.2009.10.001 19962619

[24] Hakala M, Nieminen P, Koivisto O (1994). More evidence from a community based series of better outcome in rheumatoid arthritis. Data on the effect of multidisciplinary care on the retention of functional ability. J Rheumatol.

[25] Wallace CA, Giannini EH, Huang B, Itert L, Ruperto N (2011). American college of rheumatology provisional criteria for defining clinical inactive disease in select categories of juvenile idiopathic arthritis. Arthritis Care Res.

[26] Philipp O, Valko M, Claudio L, Bassetti M, Konrad E, Bloch M, Ulrike Held P, Christian R, Baumann M. Fatigue Severity Scale (FSS, English version)*.

[27] Buysse Charles F, Reynolds Ill DJ, Monk TH, Berman SR, Kupfer DJ (1989). The pittsburgh sleep quality index: a new instrument for psychiatric practice and research. Psychiatry Res.

[28] Heyward VH, Stolarczyk LM, Heyward VH, Stolarczyk LM (1996). Applied Body composition assessment. Applied body composition assessment.

[29] Oliveira S, Ravelli A, Pistorio A, Castell E, Malattia C, Prieur AM (2007). Proxy-reported health-related quality of life of patients with juvenile idiopathic arthritis: the pediatric rheumatology international trials organization multinational quality of life cohort study. Arthritis Care Res (Hoboken).

[30] Gutiérrez-Suárez R, Pistorio A, Cespedes Cruz A, Norambuena X, Flato B, Rumba I (2007). Health-related quality of life of patients with juvenile idiopathic arthritis coming from 3 different geographic areas. The PRINTO multinational quality of life cohort study. Rheumatology.

[31] Oliveira Ramos F, Rodrigues A, Magalhaes Martins F, Melo AT, Aguiar F, Brites L (2021). Health-related quality of life and disability in adults with juvenile idiopathic arthritis: comparison with adult-onset rheumatic Diseases. RMD Open.

[32] Giani T, de Masi S, Maccora I, Tirelli F, Simonini G, Falconi M (2019). The influence of overweight and obesity on treatment response in juvenile idiopathic arthritis. Front Pharmacol.

[33] Ottaviani S, Gardette A, Tubach F, Roy C, Palazzo E, Gill G (2015). Body mass index and response to infliximab in rheumatoid arthritis. Clin Exp Rheumatol.

[34] Vallejo-Yagüe E, Burkard T, Micheroli R, Burden AM (2022). Minimal Disease activity and remission in patients with psoriatic arthritis with elevated body mass index: an observational cohort study in the swiss clinical quality management cohort. BMJ Open.

[35] Pelajo CF, Lopez-Benitez JM, Miller LC (2012). Obesity and disease activity in juvenile idiopathic arthritis. Pediatr Rheumatol.

[36] Neto A, Mourão AF, Oliveira-Ramos F, Campanilho-Marques R, Estanqueiro P, Salgado M (2021). Association of body mass index with juvenile idiopathic arthritis Disease activity: a Portuguese and Brazilian collaborative analysis. Acta Reumatol Port.

[37] Guzman J, Kerr T, Ward LM, Ma J, Oen K, Rosenberg AM (2017). Growth and weight gain in children with juvenile idiopathic arthritis: results from the ReACCh-Out cohort. Pediatr Rheumatol.

[38] Mondal R, Sarkar S, Das NK, Chakravorti S, Hazra A, Sabui T (2014). Growth of children with juvenile idiopathic arthritis. Indian Pediatr.

[39] Sandberg MEC, Bengtsson C, Källberg H, Wesley A, Klareskog L, Alfredsson L (2014). Overweight decreases the chance of achieving good response and low Disease activity in early rheumatoid arthritis. Ann Rheum Dis.

[40] Vidal C, Barnetche T, Morel J, Combe B, Daïen C (2015). Association of body mass index categories with disease activity and radiographic joint damage in rheumatoid arthritis: a systematic review and metaanalysis. J Rheumatol J Rheumatol.

[41] Baker JF, England BR, Mikuls TR, Sayles H, Cannon GW, Sauer BC (2018). Obesity, weight loss, and progression of disability in rheumatoid arthritis. Arthritis Care Res (Hoboken).

[42] Hainsworth KR, Miller LA, Stolzman SC, Fidlin BM, Davies WH, Weisman SJ (2012). Pain as a comorbidity of pediatric obesity. Infant Child Adolesc Nutr.

[43] Höfel L, Draheim N, Schramm A, Georgi M, Haas JP (2021). Rheumatic pain and chronic pain in children, adolescents and young adults. Z Rheumatol.

[44] Arnstad ED, Rypdal V, Peltoniemi S, Herlin T, Berntson L, Fasth A (2019). Early self-reported pain in juvenile idiopathic arthritis as related to long-term outcomes: results from the nordic juvenile idiopathic arthritis cohort study. Arthritis Care Res (Hoboken).

[45] Michelsen B, Fiane R, Diamantopoulos AP, Soldal DM, Hansen IJW, Sokka T (2015). A comparison of Disease burden in rheumatoid arthritis, psoriatic arthritis and axial spondyloarthritis. PLoS ONE.

[46] Taxter AJ, Wileyto EP, Behrens EM, Weiss PF (2015). Patient-reported outcomes across categories of juvenile idiopathic arthritis. J Rheumatol.

[47] George MD, Giles JT, Katz PP, England BR, Mikuls TR, Michaud K (2017). Impact of obesity and adiposity on inflammatory markers in patients with rheumatoid arthritis. Arthritis Care Res (Hoboken).

[48] Bohr AH, Pedersen FK, Nielsen CH, Müller KG (2016). Lipoprotein cholesterol fractions are related to markers of inflammation in children and adolescents with juvenile idiopathic arthritis: a cross sectional study. Pediatr Rheumatol.

[49] Grönlund MM, Kaartoaho M, Putto-Laurila A, Laitinen K (2014). Juvenile idiopathic arthritis patients with low inflammatory activity have increased adiposity. Scand J Rheumatol.

[50] Russolillo A, Iervolino S, Peluso R, Lupoli R, Diminno A, Pappone N (2013). Obesity and psoriatic arthritis: from pathogenesis to clinical outcome and management. Rheumatology (United Kingdom).

[51] Feng X, Xu X, Shi Y, Liu X, Liu H, Hou H (2019). Body Mass Index and the Risk of Rheumatoid Arthritis: An Updated Dose-Response Meta-Analysis. Biomed Res Int.

[52] Ogdie AKA (2020). Obesity and psoriatic arthritis: a narrative review. Rheumatol Ther.

[53] Radner H, Lesperance T, Accortt NA, Solomon DH (2017). Incidence and prevalence of Cardiovascular risk factors among patients with rheumatoid arthritis, psoriasis, or psoriatic arthritis. Arthritis Care Res (Hoboken).

[54] Ernste FC, Sánchez-Menéndez M, Wilton KM, Crowson CS, Matteson EL, Maradit Kremers H (2015). Cardiovascular risk profile at the onset of psoriatic arthritis: a population-based cohort study. Arthritis Care Res (Hoboken).

[55] Ruegsegger GN, Booth FW (2018). Health benefits of exercise. Cold Spring Harb Perspect Med.

[56] Hartescu I, Morgan K, Stevinson CD (2015). Increased physical activity improves sleep and mood outcomes in inactive people with insomnia: a randomized controlled trial. J Sleep Res.

[57] Verhoeven F, Tordi N, Prati C, Demougeot C, Mougin F, Wendling D. Physical activity in patients with rheumatoid arthritis. Joint, bone, spine: revue du rhumatisme. 2016;18.p. 265–70. 10.1016/j.jbspin.2015.10.002 26639220

[58] Nørgaard M, Twilt M, Andersen LB, Herlin T (2016). Scandinavian Journal of rheumatology accelerometry-based monitoring of daily physical activity in children with juvenile idiopathic arthritis. Scand J Rheumatol.

[59] Metsios GS, Stavropoulos-Kalinoglou A, Kitas GD (2015). The role of exercise in the management of rheumatoid arthritis. Exp Rev Clin Immun.

[60] Saidi O, Rochette E, Bourdier P, Ratel S, Merlin E, Pereira B (2022). Sleep in children and adolescents with juvenile idiopathic arthritis: a systematic review and meta-analysis of case-control studies.

[61] McKenna S, Tierney M, O’Neill A, Fraser A, Kennedy N (2018). Sleep and physical activity: a cross-sectional objective profile of people with rheumatoid arthritis. Rheumatol Int.

[62] Dalen Arnstad E, Glerup M, Rypdal V, Peltoniemi S, Fasth A, Nielsen S (2021). Fatigue in young adults with juvenile idiopathic arthritis 18 years after disease onset: data from the prospective Nordic JIA cohort. Pediatr Rheumatol Online J.

[63] Chen MY, Wang EK, Jeng YJ (2006). Adequate sleep among adolescents is positively associated with health status and health-related behaviors. BMC Public Health.

[64] Chaput JP, Gray CE, Poitras VJ, Carson V, Gruber R, Olds T (2016). Systematic review of the relationships between sleep duration and health indicators in school-aged children and youth. Appl Physiol Nutr Metab.

[65] Gupta P, Srivastava N, Gupta V, Tiwari S, Banerjee M (2022). Association of sleep duration and sleep quality with body mass index among young adults. J Family Med Prim Care.

[66] Krueger PM, Friedman EM (2009). Sleep duration in the United States: a cross-sectional population-based study. Am J Epidemiol.

[67] Ward TM, Yuwen W, Voss J, Foell D, Gohar F, Ringold S (2016). Sleep fragmentation and biomarkers in Juvenile Idiopathic arthritis. Biol Res Nurs.

[68] Hughes M, Chalk A, Sharma P, Dahiya S, Galloway J (2021). A cross-sectional study of sleep and depression in a rheumatoid arthritis population. Clin Rheumatol.

